# Applying new approach methodologies to assess next-generation tobacco and nicotine products

**DOI:** 10.3389/ftox.2024.1376118

**Published:** 2024-06-13

**Authors:** David Thorne, Damian McHugh, Liam Simms, K. Monica Lee, Hitoshi Fujimoto, Sara Moses, Marianna Gaca

**Affiliations:** ^1^ BAT (Investments) Ltd., Southampton, Hampshire, United Kingdom; ^2^ PMI R&D Philip Morris Products S. A., Neuchâtel, Switzerland; ^3^ Imperial Brands, Bristol, United Kingdom; ^4^ Altria Client Services LLC, Richmond, VA, United States; ^5^ Japan Tobacco Inc., R&D Group, Yokohama, Kanagawa, Japan; ^6^ Swedish Match, Stockholm, Sweden

**Keywords:** new approach methodologies (NAM), organs on a chip (OoC), human 3D tissues, next-generation products (NGP), airway models, high-content analysis, adverse outcome pathway (AOP), dosimetry

## Abstract

*In vitro* toxicology research has accelerated with the use of *in silico*, computational approaches and human *in vitro* tissue systems, facilitating major improvements evaluating the safety and health risks of novel consumer products. Innovation in molecular and cellular biology has shifted testing paradigms, with less reliance on low-throughput animal data and greater use of medium- and high-throughput *in vitro* cellular screening approaches. These new approach methodologies (NAMs) are being implemented in other industry sectors for chemical testing, screening candidate drugs and prototype consumer products, driven by the need for reliable, human-relevant approaches. Routine toxicological methods are largely unchanged since development over 50 years ago, using high-doses and often employing *in vivo* testing. Several disadvantages are encountered conducting or extrapolating data from animal studies due to differences in metabolism or exposure. The last decade saw considerable advancement in the development of *in vitro* tools and capabilities, and the challenges of the next decade will be integrating these platforms into applied product testing and acceptance by regulatory bodies. Governmental and validation agencies have launched and applied frameworks and “roadmaps” to support agile validation and acceptance of NAMs. Next-generation tobacco and nicotine products (NGPs) have the potential to offer reduced risks to smokers compared to cigarettes. These include heated tobacco products (HTPs) that heat but do not burn tobacco; vapor products also termed electronic nicotine delivery systems (ENDS), that heat an e-liquid to produce an inhalable aerosol; oral smokeless tobacco products (e.g., Swedish-style snus) and tobacco-free oral nicotine pouches. With the increased availability of NGPs and the requirement of scientific studies to support regulatory approval, NAMs approaches can supplement the assessment of NGPs. This review explores how NAMs can be applied to assess NGPs, highlighting key considerations, including the use of appropriate *in vitro* model systems, deploying screening approaches for hazard identification, and the importance of test article characterization. The importance and opportunity for fit-for-purpose testing and method standardization are discussed, highlighting the value of industry and cross-industry collaborations. Supporting the development of methods that are accepted by regulatory bodies could lead to the implementation of NAMs for tobacco and nicotine NGP testing.

## 1 Introduction

Toxicological risk assessment methods have remained largely unchanged for half a century, with the traditional default approach using high doses administered in animal studies, human exposure estimates, and the use of conservative assessment (uncertainty) factors or linear extrapolations to establish whether a given chemical exposure is deemed “safe” or “unsafe” based on human exposure as well as estimating levels of potential risk. Despite the implementation of some changes to animal testing protocols over the years, the results from new *in vitro* approaches are still judged against this process of extrapolating the adverse effects of high doses in animals to low-dose exposures in humans. Refinements to animal studies have come in the form of reducing numbers of animals or ultimately waiving certain *in vivo* tests completely (e.g., acute *in vivo* toxicity “6-pack” testing for oral, dermal, and inhalation acute lethality; eye and skin irritation; and skin sensitization) (Mansouri et al., 2021; Lee et al., 2022). This is consistent to the guiding principles (3Rs) for ethical use of animals in product testing and scientific research that has been introduced by Russel and Burch (Russell and Burch, 1959).

The 3Rs are:

• *Replacement* that seeks to use methods to avoid or replace the use of animals;
• *Reduction* using methods that allow researchers to obtain information using fewer numbers of animals in scientific studies; and
• *Refinement* using methods to reduce potential pain, suffering or distress, and enhance animal welfare.

These broadly accepted ethical principles are now embedded in the conduct of animal-based science in many countries. In 2004, the UK government funded the creation of the National Centre for Reduction Refinement and Replacement of Animals in Research (NC3Rs) with the goal that research trends do not lead to increased animal usage or suffering (Burden et al., 2015). In the U.S., the Toxic Substances Control Act (TSCA), as amended by the Chemical Safety for the 21st Century Act, directs the U.S. Environmental Protection Agency (EPA) to “reduce and replace, to the extent practicable and scientifically justified, the use of vertebrate animals in the testing of chemical substances or mixtures; and promote the development and timely incorporation of alternative test methods or strategies that do not require new vertebrate animal testing” (U.S. Environmental Protection Agency, 2023).

The challenges to use of animal models have arisen from several different applications of toxicology. Issues with animal models have been reported, including questioning the usefulness of current mouse models due to irreproducibility and poor recapitulation of human conditions and highlighting the fact that almost no animal models are validated (Justice and Dhillon, 2016; Perlman, 2016). There is also difficulty in extrapolating high doses in animal studies to low doses in humans, with 22% of all the chemicals tested in high-dose *in vivo* carcinogenicity studies being positive for cancer (Ennever and Lave, 2003). During development, 30% of drugs in Phase 1 (first use in humans) fail due to unexpected side effects or lack of efficacy, but the overall failure rate is ∼90% for drugs in Phase 1 clinical trials due to other causes such as low efficacy (Sun et al., 2022). In light of this issue, the U.S. National Academies of Science, Engineering, and Medicine (NASEM) were asked to radically rethink traditional toxicological testing methodology, based on the large numbers of chemicals already released into the environment (>10,000) that had no associated toxicological data and the potential time and cost required to implement animal tests. The primary goals for the report were: to provide as wide a coverage of chemicals, outcomes and life stages as possible; reduce the costs and time for testing; use fewer animals with less suffering; and develop more robust methods for environmental chemical assessment. It was further recommended that assays chosen should also reflect the large gains in science that have been made in the last decades such as the use of the omics technologies. The U.S. National Research Council (NRC) released a 2007 report entitled “Toxicity Testing in the 21st Century: A Vision and a Strategy” (TT21C) (National Research Council, 2007) that proposed an alternative assessment testing paradigm where virtually all routine toxicity testing would be conducted *in vitro* (in human cells or cell lines). The underlying concept was that high-throughput toxicity pathway assays could evaluate disruption in key cellular processes. Toxicological risk assessment based on results from such assays would help avoid significant perturbations of known key cellular pathways in exposed human populations. Instead, dose-response modeling of altered pathway functions could be organized based on computational systems biology models of the networks underlying each toxicity pathway (Andersen and Krewski, 2009). This concept of pathway-based approaches to risk assessment was expanded by the description of “Adverse Outcome Pathways” (AOPs). Now the challenges are translating the AOP/TT21C vision into practical tools that will be useful to those making safety decisions and determining how to provide new mechanistic data not normally reviewed by risk assessors.

Following the release of the 2007 NRC report, consortia, collaborations, and initiatives were adopted to apply TTC21 *in vitro* toxicological approaches. In the US, Toxicology in the 21st Century (Tox21) was formed as part of a federal agency consortium, bringing together the EPA, the National Toxicology Program at the National Institute of Environmental Health Science, National Institutes of Health’s National Center for Advancing Translational Sciences, and the Food and Drug Administration (Thomas et al., 2019). The goal of this program set out to develop assays to measure the pathways that lead to adverse effects in humans and develop models that can predict toxicity by using robotic technology to screen tens of thousands of environmental chemicals. Phase 1 of Tox21 involved testing 2,800 chemicals in 50 *in vitro* assays, with Phase 2 covering a further 10,000 chemicals (Attene-Ramos et al., 2013). European initiatives were developed following the ban on testing of cosmetic ingredients that came in to force in Europe 2013 (76/768EEC) (Silva and Tamburic, 2022) and continued as part of the European Union’s Horizon 2020 project (Vinken, 2020). Similar to U.S. approaches, they have goals of looking for alternative testing methods via the Safety Evaluation Ultimately Replacing Animal Testing (SEURAT) (Daston et al., 2015). More recently in 2018, the Interagency Coordinating Committee on the Validation of Alternative Methods (ICCVAM, 2018) released its roadmap for the evaluation and implementation of new approach methodologies (NAMs) to support agile validation of scientific data from TT21C-based methods to be accepted by regulatory agencies without going through full Organisation for Economic Cooperation and Development (OECD)-like validation that could take 20 years or more. At the same time, utilization of NAM approaches including extrapolating *in vitro* data to *in vivo* exposures in humans would require computational and pharmacokinetic models to predict human blood and tissue concentrations under specific exposure conditions. Unfortunately, the scientific tools needed to make these changes in toxicological risk assessment practices are still in various stages of development and qualification (ICCVAM, 2023).

Realizing this vision for the future of toxicity testing will require wide-ranging scientific discussion among stakeholders and regulators, with a potential education program to motivate a shift from animal-based toxicological tests toward an appropriate approach more firmly based on human biology. This review focuses on how such a paradigm could be applied for the evaluation of alternative next-generation tobacco and nicotine products (NGPs). The review explores how NAMs approaches can be used to assess NGPs, and will highlight key considerations, such as the use of appropriate *in vitro* model systems, the use of screening approaches for hazard identification, and test article characterization. Furthermore other considerations such as AOPs, acute verses repeated-exposures, and *in vitro* to *in vivo* extrapolation will be explored. The value of industry and cross-industry collaborations are discussed outlining the importance and opportunity for fit-for-purpose testing and method standardization. With the increased requirement of scientific studies to support regulatory approval of NGPs, use of NAMs approaches should be considered for the assessment of NGPs as part of a testing strategy.

## 2 Next-generation inhaled tobacco and nicotine products (NGPs)

Next-generation tobacco and nicotine products (NGPs) have evolved significantly over the last decade as adults who smoke seek less-harmful alternatives to conventional cigarettes. Increased global consumer uptake has driven innovation and development, which has led to greater product complexity. Toxicological risk assessment and the development of technical ingredient and product standards have enabled the development and maintenance of product quality standards and product material and ingredient quality for responsible manufacturers (Simms et al., 2019). Inclusion and adaptation of *in vitro* testing strategies can play a critical role in supporting NGP assessment, especially of inhalable products in filling the data gap in potential inhalation toxicity. NGPs not only offer the consumer an alternative choice to smoking, but these products have been reported to typically contain fewer toxicants and in lower levels compared to cigarette smoke (Margham et al., 2016; Schaller et al., 2016; Forster et al., 2018; Lu et al., 2021), offering a significant opportunity to potentially reduce the health impact of cigarette smoking on a global scale (McNeill et al., 2018; McNeil et al., 2022). This paper primarily discusses the *in vitro* testing of inhalable NGPs such as heated tobacco products (HTPs) and vapor products (also known as electronic nicotine delivery system (ENDS) or e-cigarettes (e-cigs)). While other oral products (e.g., Swedish-style smokeless tobacco products (snus) and oral nicotine pouches (ONPs) (Back et al., 2023) are increasingly available, the focus of this review is on inhalable products, so ONPs are out of scope.

HTPs utilize a specifically designed tobacco rod for use in a corresponding device that consists of a heating element, a battery, and a microprocessor controller. For HTPs to yield emissions with drastically reduced levels of harmful and potentially harmful constituents (HPHCs) as compared to cigarette smoke, the heating element should only reach temperatures below those leading to combustion of the tobacco rod (Baker, 1981; Malt et al., 2021; Lang et al., 2023; Sussman et al., 2023). The chemical composition of the resulting aerosol is typically significantly simpler than traditional cigarette smoke, with on average 90%–95% reductions in HPHC levels (Schaller et al., 2016; Forster et al., 2018). ENDS consist of a battery that powers an atomizer (microprocessor and a heating system/coil) to aerosolize an e-liquid (typically containing vegetable glycerin, propylene glycol, to United States Pharmacopoeia (USP) or European Pharmacopoeia (EP) specifications, and food-grade flavors, with or without pharmaceutical-grade nicotine). Compared to cigarette smoke, ENDS aerosols are simpler, with studied ENDS manufactured to a high-quality standard reported to yield emissions with on average 95%–99% reductions in selected HPHCs depending on the analyte assessed (Margham et al., 2016; Lu et al., 2021).

With the increased availability of NGPs and the requirement for scientific studies to support regulatory approval, NAMs may be more suitable for the assessment of such products with less complex emissions than combustible cigarettes. Recently, a number of *in vitro* toxicological approaches evaluating NGPs compared to cigarette smoke have been applied and reported (see Table 1). The use of a wide variety of different NAMs assays indicating the reduced bioactivity of both HTP and ENDs aerosols indicates the reduced harm potential of these NGPs when compared to cigarettes. However, in order to be able to compare these products against cigarettes, the test materials must be consistently generated and characterized, allowing the resulting *in vitro* exposures to be translated to human-relevant exposures.

**TABLE 1:** Summary of alternative nicotine and tobacco product characteristics and *in vitro* approaches–Examples from the literature.

Product category	HTP	ENDS
Format	• Tobacco component, battery, and heating element	• E-liquid (propylene glycol, vegetable glycerin, ± nicotine, flavorings), battery, and heating element
Consumption method	• Aerosol	• Aerosol
Chemical profile Average % reductions compared to a References cigarette	• 90%–95% (Schaller et al., 2016; Forster et al., 2018)	• 95%–99% (Margham et al., 2016)
Examples of *in vitro* data	• Significant reductions in cytotoxicity, genotoxicity, and mutagenicity (Schaller et al., 2016; Jaunky et al., 2018; Takahashi et al., 2018; Thorne et al., 2018; Ito et al., 2019; Hashizume et al., 2023) • No observed increases in tumor promotion (Crooks et al., 2018 • No impairment of endothelial cell migration and reduced effect on monocyte-endothelial cell adhesion (Poussin et al., 2016; Bishop et al., 2020) • High-content screening showed favorable differences in responses compared to cigarettes (Gonzalez-Suarez et al., 2016; Taylor et al., 2018)	• Significant reductions in cytotoxicity, genotoxicity, and mutagenicity (Thorne et al., 2016; Czekala et al., 2021; Bishop et al., 2023; Caruso et al., 2023) • No impairment of endothelial cell migration (Taylor et al., 2017) • High-content screening showed favorable differences in responses compared to cigarettes (Czekala et al., 2019)

## 3 Test articles

The laboratory assessment of NGPs involves multiple test matrices evolved from classical cigarette smoke testing using Health Canada methods to capture the various smoke fractions (Health Canada, 2024). While the test articles should most appropriately mimic the mechanism by which humans are exposed, this is not always technically feasible. The following test articles have been utilized for the assessment of NGPs: 1) aerosol captured mass (ACM), which is equivalent to classical total particulate matter (TPM) capture approaches; 2) gas vapor phase (GVP), which involves filtering the particulate material from the test article, leaving predominately the vapor phase constituents; 3) aqueous trapping approaches, where the aqueous soluble components of the aerosol are captured in an aqueous trap; 4) ACM + GVP, a combination designed to be a proxy for whole aerosol approaches by individually capturing the various phases and recombining into a single test article; and 5) whole aerosol exposure, which often requires specialized equipment to expose cells to freshly generated aerosol and maintain them at an exposure interface (Moore et al., 2023).

Whole aerosol approaches are designed to more appropriately capture the chemical-to-chemical interactions in the various phases of the aerosol and human exposures. When combined with complex co-culture or 3D human constructs, it represents the most physiologically advanced system achievable *in vitro* (Lacroix et al., 2018). However, not all assays are compatible with aerosol-generating systems (Thorne et al., 2020), and not all laboratories have the capacity to conduct aerosol-based studies. Comprehensive reviews have been published detailing the use of *in vitro* aerosolization systems and their applications (Thorne and Adamson, 2013; Klus et al., 2016; Li, 2016; Rudd et al., 2020; Cao et al., 2021; Thorne et al., 2021).

Capturing the particulate phase is a traditional, well-documented approach for cigarette smoke *in vitro* assessment (Baker et al., 2004) and has been used to assess the ACM of HTPs and ENDS (see Table 2), (Misra et al., 2014; Schaller et al., 2016; Takahashi et al., 2018; Thorne et al., 2018; Thorne et al., 2019a; Thorne et al.2019b; Iskandar et al., 2019; Miller-Holt et al., 2023). Aqueous extracts were previously used, but typically for mechanistic studies. These are also well characterized and utilized, but they are limited to the soluble fraction of the aerosol and may preferentially filter for the vapor phase constituents rather than particulates based on solubility (Bozhilova et al., 2020; Taylor et al., 2020). More recently, TPM + GVP has been used as a whole aerosol “proxy” for those occasions where the assay is not compatible with whole-aerosol methodologies or where aerosols are not standardized or available. Such approaches can capture both fractions in a 1:1 ratio and deliver more than just the particulate fraction to the cell cultures (Crooks et al., 2022). More information is available in more detail in a recent aerosol collection methods review (Smart and Phillips, 2021).

**TABLE 2:** Summary of NGP test matrices.

Test matrix	Test matrix description	Predominant fraction assessed	HTP	ENDS
ACM	Aerosol collected on a filter pad and eluted with a solvent. This approach is comparable to the generation of TPM. Traditionally used in genotoxicity testing	Particulate	X	X
Aqueous extracts* (inc. GVP partitioning)	Aerosol bubbled through an impinger to extract soluble fractions. Has been referred to as conditioned media or bubbled extracts. Traditionally used in cytotoxicity/mechanistic-based research. The particulate can be filtered, leaving just the vapor fraction	Soluble constituents (focused on vapor phase solubility using GVP)	X	X
ACM* (TPM) + GVP	Particulate matter captured and prepared and eluted using a solvent. The GVP is also captured in a bubbled aqueous solution and both fractions are recombined to create an aerosol proxy. Has been extensively used for cigarette smoke assessment, with information on NGPs recently coming online	Particulate and vapor combined in a 1:1 ratio	X	X
Whole aerosol (incl. GVP partitioning)	Freshly generated whole aerosol (or GVP based on particulate exclusion) using an *in vitro* aerosol-generating and exposure system	Compete aerosol (focused on vapor phase solubility using GVP)	X	X

ACM, aerosol collected mass; GVP, gas vapor phase; TPM, total particulate matter.

X, denotes test article has been used for *in vitro* assessments.

*Can also trap non-aqueous gas phase depending on the choice of solvent.

### 3.1 Dosimetry

A major challenge in evaluating inhalation toxicity is accurate determination of the delivered dose. In humans, breathing is a complex physical process (inhalation-pause-exhalation), and the complex anatomy of the respiratory tract makes it challenging to estimate the delivered doses to the cell surface (Alexander et al., 2008). After a substance is inhaled and deposited in the lung, particles can dissolve and absorb into the systemic/pulmonary circulation. Others are cleared from the lung by pulmonary metabolism or alveolar macrophages, and those deposited higher up in the respiratory tract are removed by mucociliary clearance (Clara et al., 2023).

The exact application of dosimetry measurements also largely depends on the exposure system being used, which should be selected based on the deposition and interaction of particles and vapors at the cell surface. Characterization of the exposure system is key to understanding the delivery of smoke/aerosol to the cell surface (Miller-Holt et al., 2023; Wieczorek et al., 2023). Ideally, dosimetry is the measure of the internal dose, or even the concentration at the molecular target (biologically effective dose) within the target cells for the chemicals of interest (Paustenbach, 2000; Escher and Hermens, 2004; Proença et al., 2021). However, directly measuring cellular dose in submerged cultures poses a significant obstacle to the application of target tissue dosimetry. For example, for nanoparticles and microparticle toxicity assessment, particularly for *in vitro* systems, due to nanoparticle agglomeration in liquids, which can alter the density of the nano particles has the ability to alter the particle transport and deposition, ultimately altering the dose response relationship (Hinderliter et al., 2010; Watson et al., 2016; Deloid et al., 2017; Thomas et al., 2018). As a consequence, the target tissue paradigm for dosimetry and hazard assessment for nanoparticles has largely been ignored in favor of using alternative indirect methods of potential or surrogate exposure such as μg particle/mL culture medium, particle surface area/mL, or particle number/mL for submerged cultures (Hinderliter et al., 2010).

Air-liquid interface (ALI) exposure is the most physiologically relevant approach to accurately determine both the dose deposited on the cell surface and the dose ultimately available to be absorbed by the cells. The most common methods are deposition of mass onto quartz crystal microbalance and the use of particle counters, photometers, or specialized gas analyzers. For a recent review of recommendations for conducting dosimetry studies in inhalable tobacco products please see the review by Miller-Holt and colleagues (Miller-Holt et al., 2023).

## 4 *In Vitro* model systems and high-content analysis

**FIGURE 1:**
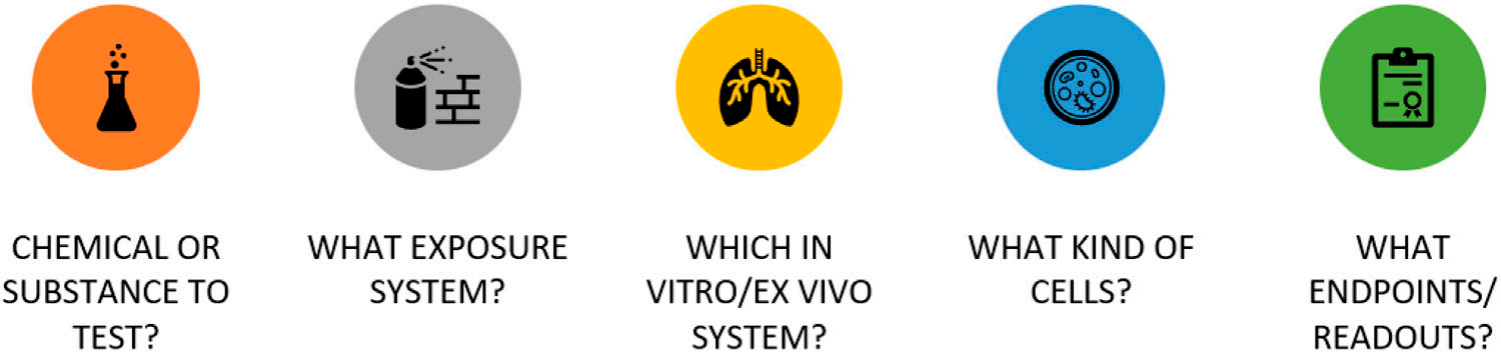
Main considerations when selecting an *in vitro* model. Reproduced with permission from Lee et al. (2022), Sharma et al. (2023). Note: 1) Test chemical and its physicochemical properties, 2) *In vitro* exposure system and aerosol characterization, 3) *In vitro* 2D/3D systems including 2D/3D, 4) cellular types and relevant tissues, and 5) assay endpoints and clinical relevance.

Animal experiments are being strongly scrutinized or entirely replaced (e.g., in the case of the cosmetics industry in certain countries) to respect the guiding principles of the 3Rs to “Replace, Reduce, Refine” in animal testing (Russell and Burch, 1959; van Meer et al., 2015). It is important that 21st Century Toxicity testing provide sufficient data that can be used for read-across purposes and ultimately to reduce animal experiments (Settivari et al., 2015). Traditional *in vitro* toxicity testing is often based on simple, single endpoints that quantify the global impact of toxicants on cells by determining increased cell permeability or reduced cell viability. However, these endpoints do not provide information on the underlying toxicological mechanism. Technological advances have facilitated the development of a series of high-content screening (HCS) *in vitro* technologies that offer a wide range of toxicological endpoints, thus increasing predictive value and complementary readouts to current regulatory toxicity testing. The goal of HCS is to provide more mechanistic information faster than traditional approaches, giving more flexibility to assess the growing diverse product landscape in a time and cost-effective manner. In general, the approaches listed below are in line with Tox21 goals (National Research Council, 2007), offering key advantages over traditional toxicity testing. Some of these NAMs have tradeoffs, but all have increased throughput, added mechanistic value, greater human and/or biological relevance, and multiplexing opportunities to maximize tissue and increase information gained. Furthermore, they provide mechanistic insights could also be used to inform AOPs (Wheeldon et al., 2020). Additionally, prior to selecting the appropriate *in vitro* models, there are a series of question(s) to be first addressed–for example, Figure 1 lists key questions in designing inhalation *in vitro* testing (Lee et al., 2022; Sharma et al., 2023).

The following summary highlights the strengths and weaknesses of *in vitro* alternatives to animal testing for inhaled toxicants relevant to tobacco and nicotine products. In particular the focus will be on screening approaches such as HCS, ToxTracker™ and MultiFlow^®^ and respiratory *in vitro* models including 2D and 3D approaches, use of *ex vivo* models and organs-on-a-chip (OoC).

### 4.1 High-content screening (HCS)

HCS enables investigative toxicity testing *in vitro* to provide knowledge about the affected biologic processes and functions. It generally refers to automated (high-throughput) microscopy, multi-parameter image processing, and visualization to extract quantitative data from cells growing in multi-well cell culture plates. HCS typically uses fluorescent imaging to trace the effect of chemicals on different toxicity pathways including oxidative stress, apoptotic cell death, DNA damage, and mitochondrial health and can be performed in a multiplexed fashion (Gonzalez-Suarez et al., 2016; Taylor et al., 2018; Czekala et al., 2019). In addition, HCS allows morphometric analysis for evaluating toxicant-induced effects on the morphology or size of cells and organelles. All these analyses can be performed with fixed cells or in a time-resolved mode by using live-cell imaging. HCS is also commonly used to monitor spatial (re)distribution of target molecules inside cells, which is pertinent to transcriptional activation following cell stress and inflammatory challenges (Czekala et al., 2019). Similar to flow cytometry, HCS enables information to be collected for multiple endpoints at an individual cell level or from an entire cell population, allowing analysis of dynamic ranges across treated cells in culture.

HCS methodologies are continuously expanding; they are now being applied to two-dimensional (2D) culture systems and three-dimensional (3D) organotypic tissue culture models (Booij et al., 2019). Artificial intelligence adds another level to HCS by allowing predictive *in vitro* toxicology analysis of new chemicals (Su et al., 2016; Lee et al., 2018).

HCS is generally perceived as a powerful *in vitro* screening technology for assessing the toxicity and efficacy of chemicals. There are also several associated limitations, especially as the technology is routinely used for screening cells growing in a 2D format:

• Lack of standardization
• Unavailability of specific tracer molecules or antibodies to stain and quantify targets of interest
• Increased data storage requirements
• Lower sensitivity than other quantitative methods (e.g., quantitative polymerase chain reaction)
• Limited applications for 3D image analysis
• Autofluorescence of test items (e.g., TPM) from cigarette smoke
• HCS of 3D organotypic ALI cultures is technically more demanding than for standard 2D culture systems
• Limited markers (e.g., cytotoxicity and H2AX for genotoxicity) have been demonstrated with whole aerosol approaches, but focusing and imaging the ALI can be problematic

Several groups have employed HCS to assess the biological impact of NGP aerosol fractions (Gonzalez-Suarez et al., 2016; Taylor et al., 2018; Czekala et al., 2019). All of these studies demonstrated the utility of HCS as a tool for NGP product assessment. They also provided *in vitro* evidence for reduced biological impacts of fractions generated from HTPs and other nicotine-containing products compared to those of cigarette smoke fractions. Moreover, complementary test methods, such as those sensitive for oxidative stress toxicity pathways (gene-expression analysis or reporter-gene assays), confirmed the trends observed by HCS. Once qualified, HCS may be applied as a standard platform for modern toxicologic analysis of NGPs.

### 4.2 ToxTracker™

ToxTracker™ (Toxys, Oegstgeest, the Netherlands) is a high-content assay that employs a series of mouse embryonic stem cell reporter gene cell-lines (Czekala et al., 2021). It consists of six green fluorescent protein (GFP) reporter gene lines plus a control wild-type line, providing readouts on biomarkers for oxidative stress (Srnx1 Nrf2 dependent and Blvrb Nrf2 independent), DNA damage (Bscl2 and Rtkn), cell stress (Btg2), and protein damage/misfolding (Ddit3). ToxTracker has been qualified against >450 known compounds and possesses ≥95% sensitivity and selectivity for both the Ames and *in vivo* micronucleus assays for mutagenicity and genotoxicity, respectively (Hendriks et al., 2016). ToxTracker could prove particularly valuable as it exhibits good/excellent concordance with classical genotoxicity testing and also offers mechanistic data on mode of action. Accordingly, ToxTracker can be a potential screening tool and/or a follow-up assay to identify mode of action in a positive *in vitro* response.

Several caveats exist for the application of ToxTracker:

• Although excellent concordance has been shown with classical toxicological approaches, the assay utilizes a mouse (not human) embryonic cell line
• As the assay is based on GFP expression, there are autofluorescence issues when using TPM from cigarette smoke (Johnson et al., 2009)
• It has not yet been combined or demonstrated to be applicable to whole aerosol approaches
• The assay is currently undergoing OECD validation, and there are few data on its use with NGPs
• Limited information exists on the assays ability to deal with complex mixtures where multiple direct and indirect acting chemicals are at play

### 4.3 MultiFlow^®^

The *in vitro* MultiFlow^®^ (Litron Laboratories, Rochester, NY, USA) genotoxicity flow cytometric assay multiplexes several biomarkers that are responsive to diverse forms of DNA damage. The multiplexed biomarkers include: 1) phosphorylation of H2AX at serine 139 to detect double-strand DNA breaks, 2) phosphorylation of histone H3 at serine 10 to identify mitotic cells, 3) nuclear p53 content as an indicator of p53 activation, 4) frequency of 8n + cells to monitor polyploidization, and 5) relative nuclei counts to provide information about treatment-related cytotoxicity (Bryce et al., 2016; Bryce et al., 2017; Dertinger et al., 2019). Some multiplexing adaptations to the methodology have been described for a more integrated genotoxicological approach, such as the combination of the MultiFlow^®^ with a flow-based *in vitro* micronucleus assays (Smart et al., 2020). The MultiFlow assay has also undergone significant inter-laboratory comparisons (Bryce et al., 2017). Eighty-four chemicals split between aneugen, clastogen, and nongenotoxin groups were collectively compared and cross-analyzed to determine inter-laboratory assay variability. Compared to historical mode of actions for the three class of chemicals, the MultiFlow assay demonstrated ≥92% sensitivity, specificity, and concordance. As a result, an excellent “training” list and established data are available to cross-reference results (Bryce et al., 2017).

Some limitations of MultiFlow include:

• The variability observed in the biomarker endpoints could confound result interpretation in less experienced laboratories. Analyzing multiplexed data requires careful interpretation and consideration.
• It has not been combined or demonstrated to be applicable to whole aerosol approaches
• Limited information exists on the assays ability to deal with mixtures where multiple direct and indirect acting chemicals are at play.

### 4.4 *In Vitro* airway models

Common *in vitro* models for studying the biological impact of inhaled toxicants on human airway epithelial cells are based on 2D cell culture systems. Tumor cell lines and immortalized primary epithelial cells (Calu-3, BEAS-2B, 16HBE14o-, NCI-H292, NCI-H441, RERF-LC-AI, and A549) grown in submerged (2D) culture conditions have been frequently used; however, they do not accurately recapitulate the native airway epithelia (Gordon et al., 2015). Although most of them can also be grown at the ALI (Haswell et al., 2010), drawbacks of using these epithelial cell lines for *in vitro* toxicity studies include their limited metabolic competency that might affect their responsiveness to toxic stimuli (Garcia-Canton et al., 2013), the absence or reduced formation of tight junctions, and their refractoriness to differentiation (Stewart et al., 2012). Non-differentiated primary epithelial cells grown in submerged conditions do not fully reflect native airway epithelia because they either lack the polarized morphology and expression of markers (ion channels) normally found in airway epithelia (Kunzelmann et al., 1996) or show altered responsiveness to solid-particle exposure relative to differentiated primary epithelial cells (Ghio et al., 2013). Due to technical convenience (propagation and possibility for higher throughput), 2D culture systems based on cell lines are most suited for screening purposes to investigate the toxicity of a greater number of test items. Transformation techniques based on hTERT/Cdk4 allowed the generation of immortalized cell lines from primary lung epithelial cells that can differentiate into mucin-producing and ciliated airway models that more closely resemble the lung epithelium (Vaughan et al., 2006).

Reconstituted 3D organotypic culture systems from primary epithelial cells grown at the ALI (Nichols et al., 2013) also replicate the cellular complexity of pseudostratified epithelium found in human airways, containing ciliated/non-ciliated epithelial cells as well as basal (progenitor) cells (Gray et al., 1996; Prytherch et al., 2011). Furthermore, they closely mimic epithelial functionality by means of cilia beating, apical mucus secretion, ion channel expression, and the presence of tight junctions (BeruBe et al., 2009). These models also match the metabolic competency of the native tissue (Iskandar et al., 2013) and are amenable to whole aerosol exposure. The last characteristic can make an important difference regarding the potential underestimation of the biological impact of complex mixtures such as cigarette smoke, because frequently used smoke extracts such as TPM or GVP do not contain the totality of cigarette smoke constituents.

Three-dimensional epithelial cells from different zones of the human aerodigestive tract are commercially available as ready-to-use ALI cultures under brand names such as EpiAirway™ (MatTek Corporation, Ashland, MA, USA) MucilAir™, and SmallAir™ (both from Epithelix Sàrl, Geneva, Switzerland). They can be cultured for weeks to months and may be used for repeated (chronic) exposure (Czekala et al., 2021). The primary alveolar test model systems is increasing in use and commercial availability, with models currently available from suppliers such as Epithelix, ImmuONE (Hatfield, UK), and Invitorlize (Belvaux, Luxembourg). The use of these models is relatively new and beyond the scope of this paper.

Even though 3D organotypic cell cultures closely resemble the native tissue, they usually lack the cellular components of the immune system. More complex model systems have been evaluated as potential candidates for routine aerosol testing (Lehmann et al., 2011; Klein et al., 2013; Marescotti et al., 2019). They use ALI conditions and combine immune cells from the innate immune system or fibroblasts with epithelial cell layers. Attempts to produce functional airway epithelial model systems originating from pluripotent stem cells are also promising and might provide additional mechanistic understanding of aerosol-induced toxicity while also allowing the generation of *in vitro* models from specific patient populations (Wong et al., 2015). No single *in vitro* model can answer every study question, so a pragmatic approach should be tailored depending upon the exact hypotheses.

### 4.5 *Ex Vivo* models and organ(s)-on-a-chip

The use of precision-cut lung slices (Behrsing et al., 2013; Sewald and Braun, 2013) and lung-on-chip model systems (Huh et al., 2010; Stucki et al., 2015) resembling additional aspects of the human respiratory tract provide opportunities for characterizing aerosol-induced toxicity *in vitro*, with an even greater relevance to native tissue and the possibility to emulate pharmacokinetics. Other approaches include using co-cultures of various organ cell types as a cell-based disease screening model (BioMap^®^) to screen drugs and NGPs for potential adverse effects (Simms et al., 2021).

*In vitro* organ models have received considerable attention in the attempt to reduce animal experiments and decrease the rate of preclinical failure associated with drug development. Two major advances in the *in vitro* research field are helping address the weaknesses of the simple models used in the past, rendering them more physiologically relevant and predictive for the effects of compounds in humans: 1) simple 2D cell culture systems are increasingly replaced by organotypic culture systems with 3D architecture, and 2) static culture systems have been switched to dynamic exposure models. The merging of these two developments has culminated in OoC technology, which combines 3D organotypic culture systems (organs) with microfluidic devices (Alepee et al., 2014; Bovard et al., 2017).

Microfluidics enable interconnection of different organ models on the same chip platform by circulating culture medium from a common reservoir. These multi-organ-on-a-chip (MOC) devices permit organ-to-organ crosstalk, which allows compounds to be tested in a more *in vivo*-like environment (Rogal et al., 2017). To date, long-term, MOC-based co-cultures of different organ combinations have been established for liver spheroids with human 3D lungs (Bovard et al., 2018) or intestine (Maschmeyer et al., 2015a), neuronal (Materne et al., 2015) and pancreatic islet (Bauer et al., 2017) tissue models, as well as a skin–intestine–liver kidney chip (Maschmeyer et al., 2015b). At least two reported OoC systems have been designed for whole aerosol exposure of 3D lung models (Benam et al., 2016; Stucki et al., 2018). However, these platforms are not compatible with smoking machines or the *in vitro* exposure instruments commonly used in the tobacco industry.

Current OoC or MOC models have potentially improved the human relevance of *in vitro* assessment of chemicals (see Table 3 for systems strengths and challenges). While there are demonstrated examples in drug candidate profiling and investigative toxicology, their application for NGPs is currently in its infancy.

**TABLE 3:** Summary of major advantages/challenges of OoC and MOC models.

Advantages of OoC- and MOC-based *in vitro* models
More physiologically relevant, as the phenotype and functionality of used 3D organotypic model systems more closely resemble the human tissue counterparts
• Organ-to-organ cross-talk (MOC)
• Mechanical forces mimicking blood flow or breathing create more physiological conditions. For instance, endothelial barrier formation is promoted upon shear stress [Buchanan et al. (2014); Ohashi et al. (2023)]
• Allow *in vitro* investigation of ADME
• Compound metabolism by a liver surrogate in the chip can be analyzed, as can its systemic toxicity/efficacy on different organs (MOC)
• Support PBPK modeling

Challenges of OoC- and MOC-based *in vitro* models
Stability of organ models in the chip
• Co-culture medium and material compatibility
• Technical challenges such as contamination and air-bubble trapping
• OoC and MOC properties depend mainly on the source of the cells used to create the model (e.g., primary cells versus iPSCs or multiple cell types in one model)
• No model standardization (standard protocols to produce, load, and culture the chips, as well as qualification of OoC and MOC functionality)
• Scaling of components (e.g., medium volume or number of cells) might not fully reflect the *in vivo* counterpart, which could lead to inaccurate PBPK
• Adsorption of test compounds to chip surfaces (common issue with PDMS-based chips)
• Most OoC and MOC platforms provide only low-throughput capacity
• Only a few systems designed for whole aerosol exposure of lung models on the chip
• High cost

ADME, absorption, distribution, metabolism, and excretion; iPSC, induced pluripotent stem cell; MOC, multi organ on a chip; OoC, organ on a chip; PBPK, physiologically based pharmacokinetic; PDMS, polydimethylsiloxane.

In summary, there are a number of established alternative *in vitro* models to animal testing that are more sophisticated than simple cell lines and suitable for investigating the biological impact of inhaled toxicants in an environment more relevant to human exposure. However, model validation remains important for ensuring acceptance by regulatory bodies. Additionally, inter-donor differences are difficult to capture in *in vitro* aerosol exposure studies, particularly with 3D organotypic ALI exposure, which is usually not sufficiently scalable to test a variety of donors (Mori et al., 2022). The development of a relevant human lung tissue model is of ultimate importance for method standardization; the *in vitro* model must have high reproducibility and clinical relevance for inhalation toxicity assessment. However, constructing an *in vitro* lung tissue model derived from human cells currently remains challenging due to the inherent complexity of the human lung and the number of different cell types involved (Petpiroon et al., 2023).

## 5 Other considerations

### 5.1 Use of adverse outcome pathways (AOPs)

An AOP is defined as “*an analytical construct that describes a sequential chain of causally linked events at different levels of biological organization that leads to an adverse health or ecotoxicological effect”* as defined by the OECD (OECD, 2023)*.* AOPs are used to help to organize the available mechanistic information relating to an adverse outcome into the key events (KEs) that are required for the adverse outcome, spanning all organizational levels of a biological system(s) (Lowe et al., 2017; Luettich et al., 2017). Key event relationships (KERs) define the relationship between a pair of KEs by showing which is up-/downstream and are supported by both biological plausibility and empirical evidence (Villeneuve et al., 2014). The use of AOPs to organize information into AOP networks has the potential to improve the use of mechanistic data and lead to improved regulatory decision making (Villeneuve et al., 2014). In this way, the use of AOPs can greatly improve the biological understanding of a particular disease process through a simplified series of events. The critical element is causality as the AOP moves from 1 KE to another (i.e., KE 1 always occurs before KE 2, etc.). AOPs can also help link biological exposures to the eventual toxic effects at the population level.

In terms of regulatory context, knowledge of disease mechanisms can guide the design of testing strategies using *in vitro* methods that can measure or predict KEs relevant to the biological effect of interest. AOPs are not chemical specific, but they link the molecular initiating event (MIE) for a chemical to the apical end point, which is the observable outcome in the whole organism and typically a clinical sign or pathological state (Burden et al., 2015). The well-considered use of AOPs could drive positive changes in toxicology testing, moving toward less reliance on making predictions based on animal models and focusing on the measurement of apical toxicity endpoints (Burden et al., 2015).

### 5.2 Acute vs repeated exposure

The vast majority of *in vitro* toxicity testing is restricted to one-time (acute) treatment of cells with a test item for a relatively short exposure duration (hours to a few days) before endpoint measurement. Such tests include escalation of the dose to a point where toxic (adverse) effects are either visible at the morphological level or quantifiable by standard cytotoxicity (viability) tests. While dose–response analyses in cell-based assays to determine acute effects on cell cultures are commonly used to rank the toxicity of different chemicals, they have several limitations: 1) the doses for inducing adverse effects are often selected based on potential toxicity hazard that are not relevant for human exposure levels (environmental or use levels); 2) therefore, the toxicity mechanisms observed *in vitro*, especially after high dosing for eliciting acute toxicity in cell-based assays also may not reflect relevant mechanisms in humans; and 3) they cannot fully predict the effects of subtoxic concentrations of a chemical when administered repeatedly (Ito et al., 2020; Czekala et al., 2021; Chapman et al., 2023). The latter is pertinent, as smoking-related diseases such as chronic obstructive pulmonary disease or cardiovascular diseases only manifest after chronic exposure to cigarette smoke (Yoshida and Tuder, 2007; Luettich et al., 2021; Farcher et al., 2023). Chronic exposure may also result in distinct adverse effects not seen after acute exposure or cause adaptive responses that are not detectable following a single treatment. Adaptations often invoke compensatory repair mechanisms elicited upon chronic stress. These considerations are also relevant for testing inhalable toxicants *in vitro* and are reflected in a number of published studies on assessing NGPs through repeated exposure of cells, as discussed below.

#### 5.2.1 Repeated exposure of lung epithelial cells to aerosol or smoke fractions

Long-term (repeated) exposure to subtoxic concentrations of cigarette smoke condensate (CSC) or total particulate matter (TPM) from 3R4F reference cigarettes for up to 12 weeks has been reported to induce an epithelial to mesenchymal transition-like phenotype in BEAS-2B cells, along with anchorage-independent growth of the cells and transient effects on both oxidative stress and DNA damage (Veljkovic et al., 2011; van der Toorn et al., 2018). These effects are only visible when cells are exposed to a much higher concentration of ACM from an NGP. Another study evaluating the short- and long-term effects of TPM on mitochondrial function in BEAS-2B cells (Malinska et al., 2018) revealed a short-term decrease in mitochondrial respiration rate after 1 week of repeated exposure, accompanied by an increase in oxidative stress markers upon 3R4F TPM treatment and cellular adaptation to stress after repeated exposure for 12 weeks. In case of ACM from a NGP, the concentrations required to elicit these effects were again much higher than those derived from reference cigarettes. A similar study on the effects of prolonged exposure to 3R4F cigarette smoke extract (CSE) on BEAS-2B cells reported increased mitochondrial capacity (Hoffmann et al., 2013). These contradictory results may be explained by the different smoke extract used, as well as the applied dose and overall duration of exposure. In a different study, the long-term effects of 1–16 weeks of exposure to nicotine or CSE from 3R4F has been analyzed in BEAS-2B cells (Stabile et al., 2018). In this repeated exposure study, distinct effects of CSE *versus* pure nicotine were observed when analyzing cell viability and other cellular parameters, including nerve growth factor/receptor gene expression.

Repeated exposure experiments were also extended to differentiated primary human bronchial epithelial organotypic cell cultures, which showed cumulative effects on inflammatory responses and tissue morphology after 1-month repeated exposure to 3R4F TPM (Ito et al., 2018). Interestingly, repeated exposure of bronchial epithelial organotypic cells to 3R4F CSE or TPM during the differentiating phase of the culture had profound effects on cell composition when measured after 28 days; although the number of ciliated cells decreased, those of Clara and goblet cells increased (Haswell et al., 2010; Schamberger et al., 2015).

#### 5.2.2 Repeated exposure of lung epithelial cells to whole aerosols

Several studies evaluated the short-to-long-term effects of subtoxic concentrations of whole cigarette smoke on 3D organotypic ALI tissue cultures reconstituted from human primary epithelial cells, which were tested as either mono- or co-cultures with fibroblasts. The authors reported transition of normal bronchial epithelia towards a metaplastic phenotype and fewer cilia-bearing cells after repeated exposure (4–13 times) to mainstream 3R4F smoke (Aufderheide et al., 2015; Aufderheide et al., 2017). This suggests that the effect on cilia is caused by volatile organic constituents present in mainstream smoke. Similarly, reductions of ciliated, mucus-producing, and club cells were observed when differentiated immortalized primary normal human bronchial epithelial cells were repeatedly exposed to cigarette smoke or e-cigarette vapor (Aufderheide and Emura, 2017). In that study, metaplastic areas positively stained for the basal cell marker cytokeratin-13 were also identified after exposure to both cigarette smoke and e-cigarette vapor. In a co-culture model with ‘omics’ analysis using human 3D bronchial tissue cultures combined with human fibroblasts, the central carbon metabolism in relation to oxidative stress response was perturbed after 21 days of repeated exposure to 3R4F whole smoke; additionally, the epidermal growth factor receptor was identified as a key regulator of perturbed processes (Ishikawa et al., 2019). Repeated exposure (21 days) of differentiating bronchial epithelial 3D organotypic cultures to 3R4F whole smoke, in contrast to repeated exposure to CSE, did not result in an increase in goblet cells (Ishikawa and Ito, 2017).

### 5.3 *In vitro*-to-*in vivo* extrapolation (IVIVE)

While *in vitro* experiments have many advantages over *in vivo* testing in terms of increased human relevance as presented above, using *in vitro* data for the purpose of risk assessment (e.g., quantifying the margin of safety) has many challenges, including uncertainty around how to interpret and link exposure or dose. Quantitative *in vitro*-to-*in vivo* extrapolation (IVIVE) is an emerging NAM-based computational tool to facilitate this process, generally referred as an extrapolation of (human tissue-derived) cellular (*in vitro*) data to predict the exposures or outcomes in humans (*in vivo*). IVIVE implies certain quantitative extrapolations of “dose (or exposure)” and “response” to enable *in vitro*-based toxicological risk assessment (Zhang et al., 2021; Hines et al., 2022; Zhang and Wright, 2022). Dose extrapolation is necessary to estimate the human exposure or intake level (e.g., daily human equivalent dose) of individual toxicants, commonly by computational (e.g., physiologically-based pharmacokinetic [PBPK]) models and *in silico* and/or human-based *in vitro* data. The human equivalent dose can be extrapolated back to the corresponding exposure levels (commonly called “reverse” dosimetry) and compared to measured (environmental) exposures to evaluate the safety margin or guide regulatory safety decision-making. Therefore, one may first define and characterize the *in silico* or *in vitro*-based dose–response relationship, and the relevance of the MIEs or KEs can be selected based on the AOPs of interest. Then, using the *in vitro* dosimetry and disease-relevant response data and with the help of computational modeling, IVIVE enables “human-relevant” safety assessment, moving away from a need for conventional animal toxicity data.

While the concept of IVIVE is gaining interest in scientific communities (Bell et al., 2018; Chang et al., 2021; Hines et al., 2022), it is still an emerging field with limited application to regulatory risk assessment, especially for tobacco and nicotine products. The early successes—mostly from pharmaceutical and environmental safety assessment—typically involve single chemicals, although concerted effort is being made to apply IVIVE to mixtures (Chang et al., 2021; Zhang et al., 2021; Zhang and Wright, 2022). Many of the challenges in using IVIVE for tobacco or nicotine products are similar, including uncertainty in dose selection and quantification (which individual toxicants should be modeled as is or as mixtures in the complex and dynamically unstable aerosol mixtures) and the target test systems (which cellular/tissue systems should be used to reflect what clinically relevant outcomes at cellular levels). Additionally, there is a lack of chemical or mixture-related *in vitro* data that can be used for IVIVE model development and the independent verification of modeling outcomes.

## 6 Summary

**FIGURE 2:**
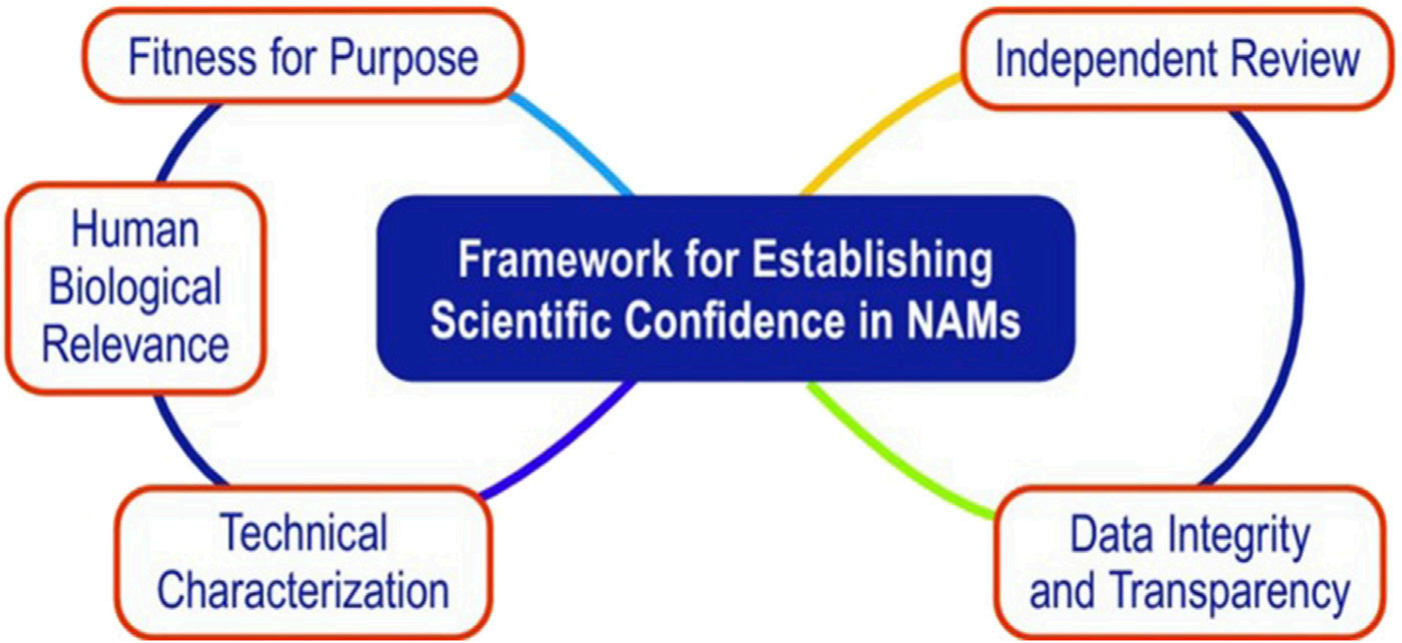
Key considerations in developing and qualifying NAM tools. Reproduced with permission from van der Zalm et al. (2022). Note: the figures illustrates the five inter-connected elements that are essential in establishing scientific confidence in NAM applications.

NAMs are gaining traction as chemical toxicity and biologically relevant assessment tools based on *in vitro* and *in silico* (computational) methodologies that support 3R *in vivo* animal testing traditionally necessary for risk assessment. Significant progress has been made toward the adoption of NAMs for human health and environmental toxicity assessment, although they are not yet fully embraced for routine use in regulatory decision making. This review summarized the current NAMs in use by major tobacco and nicotine product manufacturers. The NAMs were chosen in line with the concepts/criteria displayed in Figure 2, with a focus on the human relevance of NAMs and their potential ability to model and predict key elements of human disease processes.

As part of the effort to promote NAM applications for use in tobacco and nicotine products, two symposiums were held during the annual Cooperation Centre for Scientific Research Relative to Tobacco (CORESTA) Smoke Science and Product Technology conferences in 2021 and 2023 to introduce the concepts and potential application of NAMs for evaluating NGPs (Lee et al., 2022; Lee et al., 2023). Many of the promises are tangible based on successful case examples demonstrating that NAMs can be a pragmatic and effective approach in terms of cost, time, and resources, in addition to offering enhanced sensitivity for predicting human-relevant health impacts. At the same time, there are ample opportunities to increase confidence in NAM context of use and standardization. Finally, clarity on the degree of validation/qualification by regulatory bodies is required. This last point is essential before NAM-based risk assessments achieve full legitimacy for regulatory risk assessment.
