# Corrigendum: Applying new approach methodologies to assess next-generation tobacco and nicotine products

**DOI:** 10.3389/ftox.2024.1460271

**Published:** 2024-07-19

**Authors:** David Thorne, Damian McHugh, Liam Simms, K. Monica Lee, Hitoshi Fujimoto, Sara Moses, Marianna Gaca

**Affiliations:** ^1^ BAT (Investments) Ltd., Southampton, Hampshire, United Kingdom; ^2^ PMI R&D Philip Morris Products S. A., Neuchâtel, Switzerland; ^3^ Imperial Brands, Bristol, United Kingdom; ^4^ Altria Client Services LLC, Richmond, VA, United States; ^5^ Japan Tobacco Inc., R&D Group, Yokohama, Kanagawa, Japan; ^6^ Swedish Match, Stockholm, Sweden

**Keywords:** new approach methodologies (NAM), organs on a chip (OoC), human 3D tissues, next-generation products (NGP), airway models, high-content analysis, adverse outcome pathway (AOP), dosimetry

In the published article, there was an error in the **Conflict of interest** statement. The **Conflict of interest** statement was displayed as “Authors DT and MG were employed by BAT (Investments) Ltd. Author LS was employed by Imperial Brands. Author KML was employed by Altria Client Services LLC. Author HF was employed by Japan Tobacco Inc., R&D Group. The remaining authors declare that the research was conducted in the absence of any commercial or financial relationships that could be construed as a potential conflict of interest.”

The correct **Conflict of interest** statement appears below.

